# Influence of Processing Parameters on the Thread and Spline Synchronous Rolling Process: An Experimental Study

**DOI:** 10.3390/ma12101716

**Published:** 2019-05-27

**Authors:** Da-Wei Zhang, Bing-Kun Liu, Sheng-Dun Zhao

**Affiliations:** School of Mechanical Engineering, Xi’an Jiaotong University, Xi’an 710049, China; lbk1004638469@stu.xjtu.edu.cn (B.-K.L.); sdzhao@mail.xjtu.edu.cn (S.-D.Z.)

**Keywords:** thread and spline synchronous rolling, synchronous rolling experiments, outside diameter, minimum diameter, pitch

## Abstract

The thread and spline synchronous rolling (TSSR) process is a new developed rolling process, which can form the different profiles simultaneously in the process and can ensure the consistency of the relative position of different profiles of parts. However, the multi-meshing motions are intercoupling and the multi-deformation characteristics are intercoupling during the forming process. It can easily result in dimension overshoot, and even does not make the synchronous rolling process go smoothly. Exploring the influence of controllable processing parameters on the synchronous rolling process, especially the geometric parameters of rolled parts, is helpful to determine the parameters and control the size error for a smooth rolling process. Thus, in this paper, the effects of controllable geometric parameters and motion parameters such as billet diameter, radial feed-in speed, and rotational speed of synchronous rolling die on the TSSR process have been studied. The synchronous rolling experimental scheme was determined using an orthogonal experimental design method, and the geometric parameters of different tooth profiles of rolled parts were measured and analyzed. The experimental results indicated that: the uncoordinated meshing movement between different tooth profiles is more likely to cause tooth error of the splined section of the part; variations of the processing parameters are more likely to cause fluctuations in the size of the splined section of the part, and change of the billet diameter mainly affects the outside diameter of the threaded and splined sections, and the threaded and splined pitches are mainly affected by the motion parameters of the synchronous rolling die; the motion parameters of the rolling die should be matched and the lower rotational speed needs to match the lower radial feed-in amount per revolution; the ideal dimensional accuracy can be obtained by using an appropriate processing parameter combination, for example, the pitch error of the splined section of the part is less than 0.5 μm under one set of experimental conditions in this paper.

## 1. Introduction

Shaft parts which have thread and spline (or gear) coaxially have been widely used in automotive transmission systems [1,2], planetary roller screw [3,4], and so on, and they are a key load-bearing part. Cold rolling process can improve the strength and wear resistance of rolled thread or spline [5], and the thread and spline synchronous rolling (TSSR) process, where the thread and spline (or small module gear) are formed by one process, provides an efficient way for plastic forming of such a part. However, the multi-meshing motions are intercoupling and the multi-deformation characteristics are intercoupling during the forming process. This can easily result in dimension overshoot, and it does not make the synchronous rolling process go smoothly. Therefore, it is necessary to explore influence of processing parameters on the synchronous rolling process and the method of process control.

Spline rolling or thread rolling is widely used as a non-cutting process to manufacture shafts. However, only a splined or threaded profile can be formed in the rolling process. The implementation work of the TSSR process was carried out based on cross rolling with radial feed-in in a study [1], where the different profiles were formed by the developed synchronous rolling under the proper die design and process control. By considering the phase characteristic among rolling dies, the die structure of TSSR was investigated in theory [2,6]. On the basis of this research, a TSSR die manufacturing method integrating the phase adjustment among rolling dies and the adjustment of relative position between the threaded section and the splined section of a synchronous rolling die was developed [7]. Then, the part with acceptable visible profiles was successfully rolled using the manufacturing TSSR die. Thus, the theoretical feasibility and the operation of TSSR have been proven. However, it appears there are insufficient reports of detailed studies with respect to the dimensional accuracy of a synchronous rolled part. The feasibility of an industrial operation for TSSR needs to be further proven.

The meshing motion of the threaded section dominates the motion during the TSSR process. The variation of center distance between the workpiece and the rolling die leads to mismatching rotational speed between the threaded section and the splined section, and therefore may result in profile error. On the basis of the motion model [8] of thread rolling with varied center distance and the motion model [9] of spline rolling with varied center distance, the theoretical study on pitch error of splined section during the TSSR process was carried out [10]. The results showed that motion parameters of rolling die have a significant influence on the pitch error. However, the experimental evidence is insufficient.

**Figure 1:**
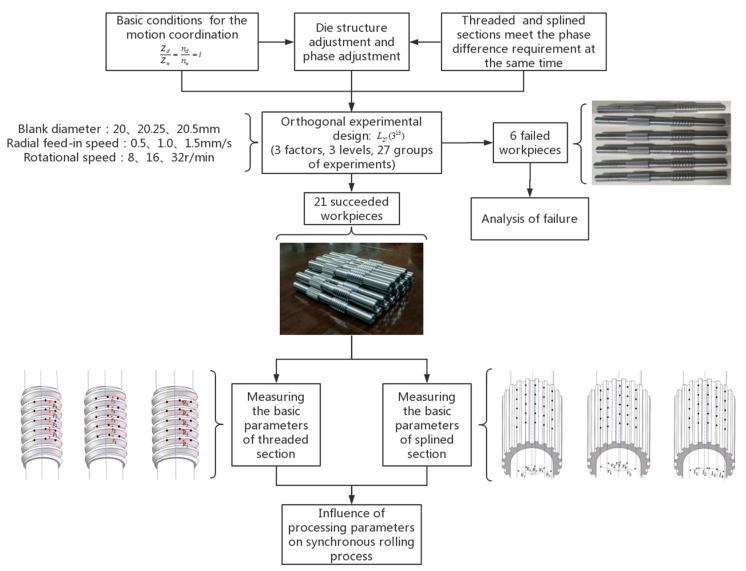
Flowchart of research in the present study.

Thus, the controllable processing parameters such as billet diameter (*d*), radial feed-in speed (*v*), and rotational speed (*n_d_*) were selected as experimental factors, the experimental scheme was determined by orthogonal experimental design method, and 27 synchronous rolling experiments were carried out. The conditions for successful synchronous rolling were analyzed and the dimensions of the threaded section and the splined section were measured, respectively. Then, the influences of the processing parameters on dimensional accuracy were investigated. The results provided a basis for the determination of optimum processing parameters. Figure 1 illustrates the research steps in the present study, which describes the theorical basis of synchronous rolling, the design of orthogonal experiments, the experiment and experimental results, and objective parameters.

## 2. Thread and Spline Synchronous Rolling Process

### 2.1. Description of Synchronous Rolling Process

#### 2.1.1. Processing Principle

**Figure 2:**
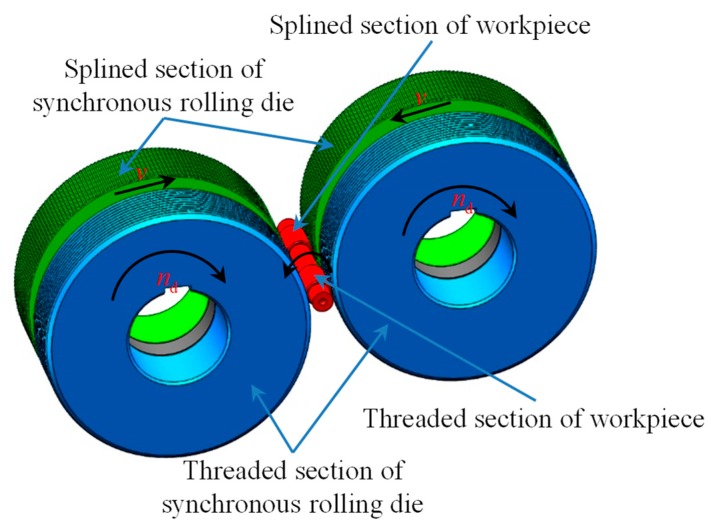
Synchronous rolling process with two round dies.

Figure 2 illustrates the synchronous rolling process with two rolling dies. The TSSR process is developed based on thread and spline rolling processes with radial feed-in, which is similar to the thread or spline rolling process, except the die structure is different. The TSSR die is composed of a threaded section and a splined section, and at the same time, the die should be able to meet the motion coordination of the threaded section and the splined section during the TSSR process and the phase difference requirement among rolling dies before rolling [1,2]. The geometric parameters of the threaded section and the splined section should meet the basic conditions Equation (1) for the motion coordination [1].

(1) $$\frac{Z_{d}}{Z_{w}}=\frac{n_{d}}{n_{w}}=i$$

where, $Z_{w}$ and $Z_{d}$ are the teeth of the splined section for the formed part and the rolling die, of the roller, respectively. $n_{w}$ and $n_{d}$ are the start of the threaded section for the formed part and the rolling die, respectively. *i* is the ratio of relationship between the roller and the formed workpiece, and the value is the ratio between the teeth or the starts of the rolling die and formed part.

The phase difference of the TSSR die before rolling is relatively difficult to adjust. In some cases, it is necessary to adjust the die structure of the TSSR die. Therefore, it is not suitable to use more than two rolling dies in the TSSR process [2]. The principle of synchronous rolling process using two dies is described as follows: a pair of synchronous rolling dies are mounted on the two drive spindles, the dies are made of the threaded and the splined sections; the two spindles are synchronously rotated in the same direction (rotational speed *n*_d_), the two spindles simultaneously perform the radial feed-in motion (feeding speed *v*), and the workpiece rotates in the opposite direction; and the threaded splined sections on different portions of the workpiece are rolled at the same time. 

#### 2.1.2. Characteristic of the Process

The TSSR can form the threaded and splined sections in one process, and it not only reduces the rolling time effectively, but also ensures the stable relative position between the threaded and splined sections. The meshing motion of the threaded section during a synchronous rolling process can promote the rotation of the workpiece and improve the dividing of splined section [1]. In addition, theoretical analysis [10] and experimental research [7] indicated that the meshing motion of the threaded section dominates the rotation during the TSSR process. Therefore, the threaded section of synchronous rolling dies should contact the workpiece first, and then the splined section contacts the workpiece.

**Figure 3:**
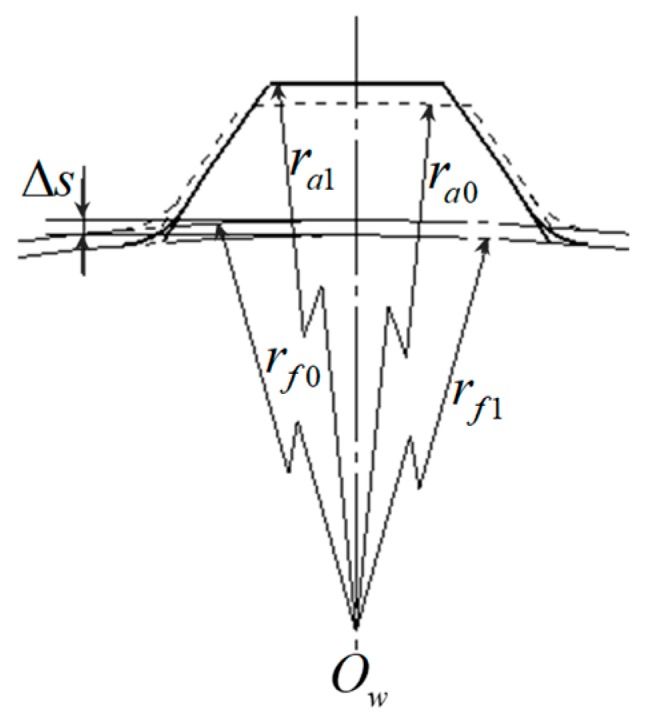
Sketch of compression amount (taking splined profile as an example) [11].

During the rolling process using *N* rolling dies to form complex profiles (such as threads, splines, gears, etc.) the rolling dies feed-in radially, and the center distance changes continuously. The same deformation area, in other words, the contact area between the workpiece and one rolling die, will be contacted and compressed by the next rolling die after the workpiece is rotated by 1/*N* revolution. A parameter such as compression amount (decrement Δs) is defined to reflect the degree of deformation of the same area during one rolling unit which is between the rolling die contact with this area and when it separates from this area. The Δs is the difference root radius of spline or thread before the rolling die contacts the workpiece and the root radius of spline or thread after the rolling die separates from the workpiece, as shown in Figure 3. When it reaches the final rolling position, the radial feed-in motion of the rolling die stops. 

**Figure 4:**
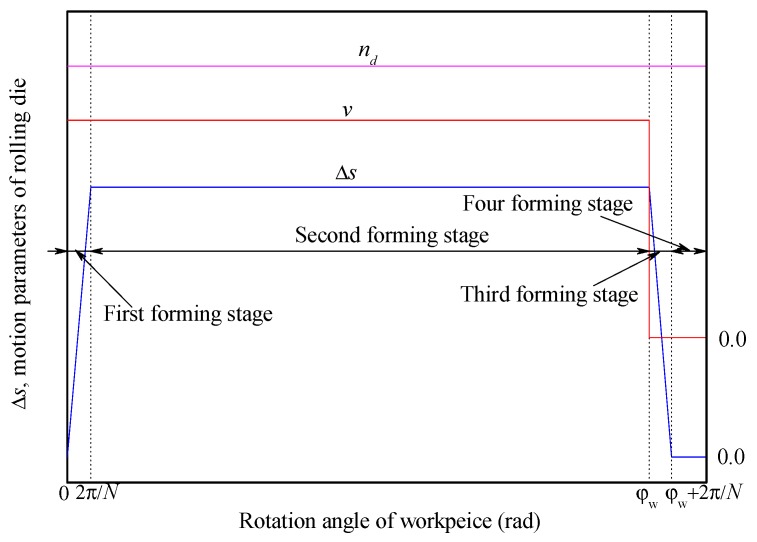
Forming stages divided according to compression amounts.

According to the change of the compression amount during the rolling process, the spline rolling process using two rolling dies is divided into four stages [9,12]. Thus, it can be further inferred that the complex profile rolling process using *N* rolling dies, such as the thread rolling process and spline (gear) rolling process, can be divided into the following four stages (shown in Figure 4), and the variation of compression amount (Δs) and related processing parameters are shown in Figure 4.

During the complex profile rolling process, the radial feed-in speed and the rotational speed of rolling die are constant in general, and the forming process can be divided into four stages, and each stage has the following characteristics:

The first stage is from the beginning of the rolling die contact with the workpiece (i.e., workpiece beginning to rotate) to 1/*N* revolution of the workpiece rotated. At this stage, the Δs is gradually increased from zero to the value of the compression amount at the stable rolling stage. The rolling die feed-in radially and rotates, the radial feed-in amount of the rolling die increases linearly, and the center distance between the workpiece and the rolling die keep decreasing.

The second stage is from the 1/*N* revolution of the workpiece rotated to the rolling die reaching the final rolling position. At this stage, the Δs remains unchanged at a stable value, and the rolling die feed-in radially and rotates. The feed-in amount of the rolling die increases linearly and increases to a maximum, as well as the center distance between the workpiece and the rolling die reduces continuously and reduces to a minimum. 

The third stage is from the rolling die reaching the final rolling position to the 1/*N* revolution of workpiece rotated again. At this stage, the Δs gradually reduces from the stable value to zero and the rolling die only rotates. The radial feed-in amount of the rolling die is constant, and the center distance between the workpiece and the rolling die is constant.

The fourth stage is from the end of the third stage to the end of the whole rolling process, which is a finishing rolling stage. At this stage, the Δs is zero and the rolling die only rotates. The radial feed-in amount and the center distance are unchanged.

During the synchronous rolling process, the deformation of the threaded section or the splined section has little influence on the axial adjacent region, and this does not affect the deformation of the adjacent splined section or threaded section [13]. Therefore, the rolling processes of the threaded section and the splined section can also be divided into the above four stages, but the first and second forming stages of the threaded section and the splined section may not coincide on the time axis, and the first forming stage of splined section may be incomplete. The compression amount Δs is determined by the rotating speed and radial feed-in speed of the rolling die, and it can be used as a comprehensive indicator of the motion parameters of the rolling die. The Δs in the steady rolling stage (second forming stage) can be approximately determined by Equation (2), where *i* is calculated by Equation (1).

(2) $$\Delta s\approx \frac{2\pi }{iN\omega_{d}}v=\frac{60}{iNn_{d}}v$$

where, the $\omega_{d}$ and $n_{d}$ are the angular velocity and the rotational speed of rolling die, respectively; *v* is the radial feed-in speed of rolling die; *N* is the number of rolling die and *N* = 2 is often adopted in the synchronous rolling process.

### 2.2. Experimental Scheme and Experimental Result

#### 2.2.1. Design of Experimental Scheme

The controllability of the parameters, cost and period, should be comprehensively considered when choosing the experimental factors. For example, in order to reduce the experimental cost, geometric parameters of the workpiece and the rolling die were not selected as experimental factors. In order to facilitate the adjustment of the parameters, the blank diameter ($d_{Z}$) of the forming zone, the radial feed-in speed (*v*) and the rotational speed ($n_{d}$) of the synchronous rolling die were selected as the experimental factors. Three levels for the selected three parameters were determined according to equipment parameters and theoretical blank diameter, as shown in Table 1.

Considering the interaction among experimental factors, an orthogonal experimental design array $L_{27}(3^{13})$ [14] was adopted according to the experimental factors and their levels, as listed in Table 2. Factors A, B, and C are assigned to 1st, 2nd, and 5th columns, respectively. The interaction columns of factors A and B are 3rd and 4th columns, the interaction columns of factors A and C are 6th and 7th columns, the interaction columns of factors B and C are 8th and 11th columns, and the remaining columns are blank.

#### 2.2.2. Experiment of Synchronous Rolling Process

In order to ensure that the threads or splines (gears) rolled by different rolling dies can be well connected, the dies before thread or spline rolling must satisfy a certain phase difference [2,6]. The threaded and splined sections of the TSSR die must meet both of the phase difference requirements before thread and spline rolling, so there is a clear requirement for the relative position between the threaded section and the splined section of the TSSR die [2]. In fact, if the threaded and splined sections of the TSSR die are considered as a whole to be manufactured, it is difficult to achieve phase coordination of the different tooth profiles of the TSSR die before rolling. According to the characteristics of the thread and spline tooth types and the synchronous rolling process, a manufacturing method combining phase adjustment before rolling and structural adjustment of the TSSR die was proposed [7]. That is to say, the threaded section and the splined section of TSSR die are manufactured, separately, and the adjustment of relative position between the threaded section and the splined section and the phase coordination before rolling are realized by adjusting the thickness of the spacer/pad between the threaded section and the splined section of TSSR die.

In this study, this method of manufacture and adjust phase is also adopted. Table 3 lists the parameters of the TSSR die used in the synchronous rolling experiment. The servo direct drive CNC rolling machine Z28K-25 was used for 27 sets of the thread and spline synchronous rolling.

The material of blank used in synchronous rolling experiments was AISI 1045 steel, and its chemical constituents are listed in Table 4 according to Chinese standard [15]. The mechanical characteristics during the plastic deformation of AISI 1045 steel can be described by Equation (3) according to the uniaxial tension in a previous study [16].

(3) $$\sigma =1450{\left(0.0132715+\varepsilon \right)}^{0.2817}$$

where, the σ and ε are the stress and the plastic strain, respectively.

#### 2.2.3. Experimental Results

The 27 TSSR experimental results fall into two types according to the macroscopic shape. Therefore, 21 experimental workpieces were with good shape and six experimental workpieces were with notable profile defects, as shown in Table 5. The thread profile connects well but the spline rolled by different rolling dies does not connect well for all six failed workpieces. Some thread of the six failed workpieces are not fully formed, because the synchronous rolling process will stop after the spline teeth are distorted. This provides further evidence that the thread meshing dominates the rotating motion and mismatching rotation between the threaded and splined sections will result in a profile error for the splined section.

**Figure 5:**
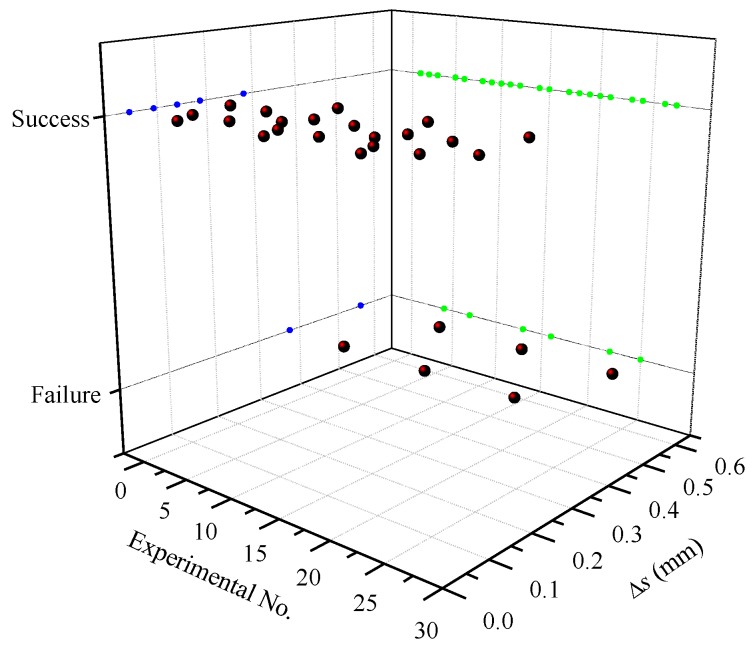
Synchronous rolling results under different compression amounts.

The compression amount Δs is a comprehensive indicator of the rotational speed and the radial feed-in speed of the synchronous rolling die. According to the Equation (2), the Δs decreases with increases in the rotational speed of the synchronous rolling die, and decreases with decreases the feed-in speed of the synchronous rolling die. When the rotational speed is 8 r/min and the feed-in speed is 0.5, 1, 1.5 mm/s, the compression amounts calculated by Equation (2) are 0.1875, 0.375, and 0.5625 mm, respectively. The compression amount at the feed speed of 1 mm/s and 1.5 mm/s is twice and three times the feed-in speed of 0.5 mm/s. The compression amounts under the experimental conditions of 27 groups were approximately estimated, and Figure 5 illustrates the relationship between the compression amounts and the forming results. The compression amounts for the successful synchronous rolling experiments are all less than 0.3 mm.

## 3. Measuring Instrument and Data Processing

**Figure 6:**
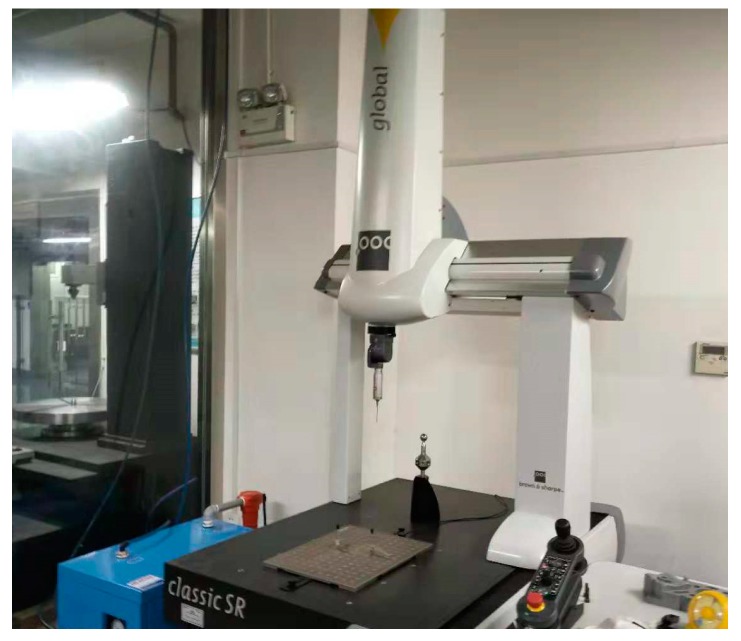
Three-coordinate measuring instrument (Global classic SR0575).

For the measurement of the size of the splined and threaded sections, the measurement is carried out using a three-coordinate measuring instrument (Global classic SR0575) manufactured by Hexagon (Stockholm, Sweden), as shown in Figure 6.

**Figure 7:**
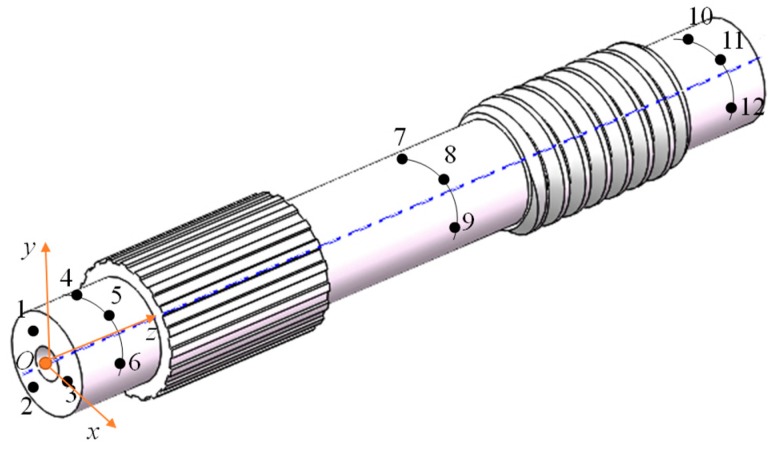
Position and coordinate system.

In order to facilitate the processing of the measured data, the coordinate origin and coordinate axis of the workpiece should be determined before the measurements of the threaded and splined sections. Figure 7 illustrates the positioning method. First, three points (points 1, 2, 3) on one end are taken to define the reference plane, then, nine points on the cylinder (points 4, 5, 6, 7, 8, 9, 10, 11, 12) are taken to define the cylinder, and then the coordinate system O-xyz is established and the z-axis coincides with the axis of cylinder.

**Figure 8:**
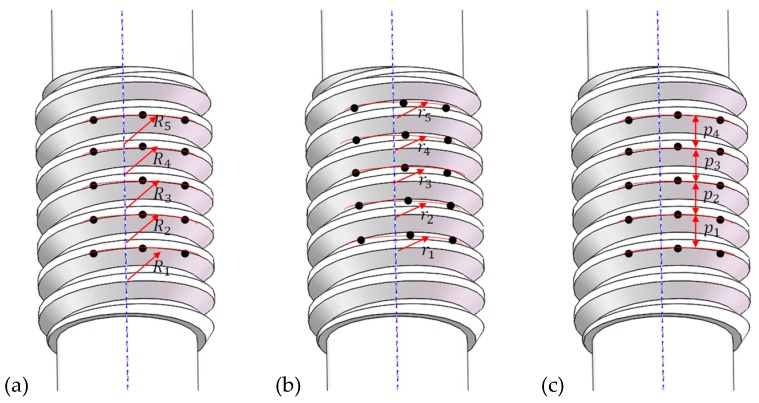
Measuring method for basis parameters of the threaded section: (**a**) outside diameter, (**b**) minor diameter, (**c**) pitch.

For the threaded section, the major/outside diameter, minor diameter, and pitch were measured. Figure 8a illustrates the measuring method for the major diameter. Three points are defined at the edge of thread crest and a circular arc is determined by the three points, the diameter of the arc is obtained and five thread crests were chosen to take the points. The average of the five diameters is defined as the major diameter of the threaded section. The measuring method of minor diameter is similar to that of the major diameter, as shown in Figure 8b. Three points are defined in the thread root region and a circular arc is determined by the three points, the diameter of the arc is obtained and five thread root regions were chosen to take the points. The average of the five diameters is defined as the minor diameter of the threaded section.

Figure 8c illustrates the measuring method of pitch for the threaded section of the synchronous rolling workpiece. Three points are defined at the edge of thread crest and a circular arc is determined by the three points, and five successive thread crests were chosen to take the points. Then, and the distance between adjacent arcs is taken, and the average of the five distances is the pitch of the threaded section.

**Figure 9:**
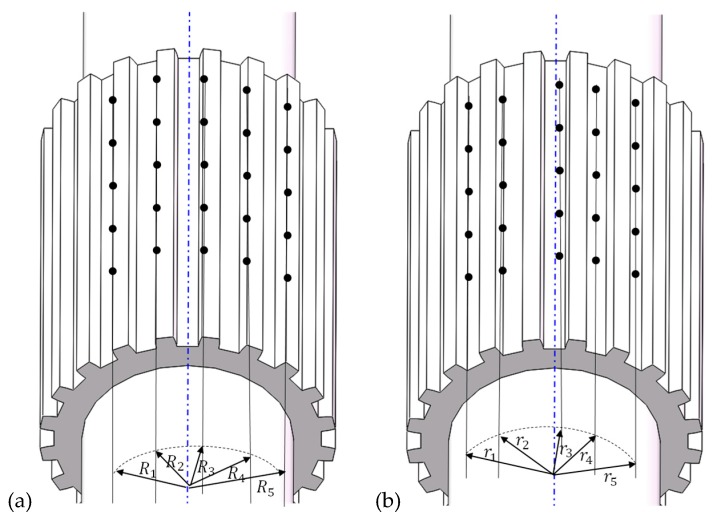
Measuring method for basis parameters of the splined section: (**a**) outside diameter, (**b**) root diameter.

For the splined section, the outside diameter (i.e., diameter of addendum circle), the root diameter (i.e., diameter of dedendum circle), and the pitch were measured. Figure 9a illustrates the measuring method of the outside diameter. Five points are defined at the edge of addendum, and a straight line is determined by the five points, five addendums of spline were chosen to take the points. The distance between the line and the axis is the radius, and then the diameter can be obtained. The average of the five diameters is defined as the outside diameter of splined section. The measuring method of root diameter is similar to that of the outside diameter, as shown in Figure 9b. Five points are defined at spline root, and a straight line is determined by the five points, five spline roots were chosen to take the points. The distance between the line and the axis is the radius, and then the diameter can be obtained. The average of the five diameters is defined as the root diameter of the splined section.

**Figure 10:**
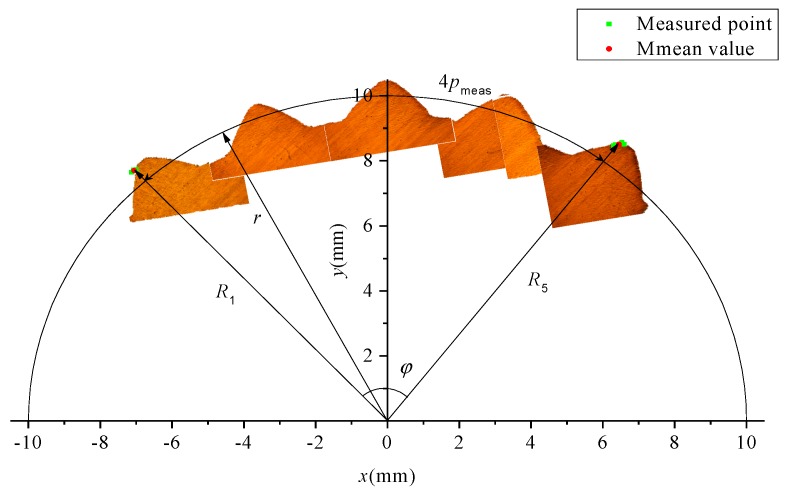
Measuring method of pitch of splined section.

Figure 10 illustrates the measuring method for the pitch of the spline. Here, the low-magnification photos for single tooth are pieced together by overlapping the iterative area. Then, the radial position of teeth in the coordinate system was determined by the measured outside and root diameters. Five lines were defined when measuring the diameter of the addendum circle. The distance between the line at the edge, and the other edge, and the axis are signed as *R*_1_ and *R*_5_, respectively, as shown in Figure 9a and Figure 10. The distance (i.e., the chord length) between the two lines is signed as *l*. According to the cosine theorem, the center angle, Equation (4), corresponding to the chord length can be obtained.

(4) $$\varphi =\arccos\frac{R_{1}^{2}+R_{5}^{2}-l^{2}}{2R_{1}R_{5}}$$

The tooth shape observed using a microscope and ten points of the coordinates measured by the three coordinate measuring instruments (in *O-xy* coordinate system) on the two lines at both edges are shown in Figure 10. It can be seen from the figure that the measuring points are all at the edge of the top of tooth, and the relative spatial position for average coordinate (position) of the measuring points at the edge is the same as that at the other edge. Therefore, the arc length corresponding to the center angle φ on the reference circle should be the pitch of four teeth, and therefore the pitch for single tooth on the reference circle can be expressed by Equation (5).

(5) $$p_{\mathrm{meas}}=\frac{\frac{mZ_{w}}{2}\varphi }{4}=\frac{mZ_{w}}{8}\varphi$$

The measured basic parameters of the threaded and the splined sections are signed as *X*_meas_. The theoretical parameters determined according to conjugate surface (line) of rolling die are signed as X_theory_. In order to evaluate the results, the absolute value of the difference between the two values is taken as the error term for analysis, as shown in Equation (6). The errors discussed below are all the values determined by Equation (6), excepting special declaration.

(6) $$Error=\left|X_{\mathrm{meas}}-X_{\mathrm{theory}}\right|$$

The measured data for threaded and splined sections of the TSSR parts have been listed in Table 6.

## 4. Results and Discussion

### 4.1. Basis Parameters of Threaded Section

**Figure 11:**
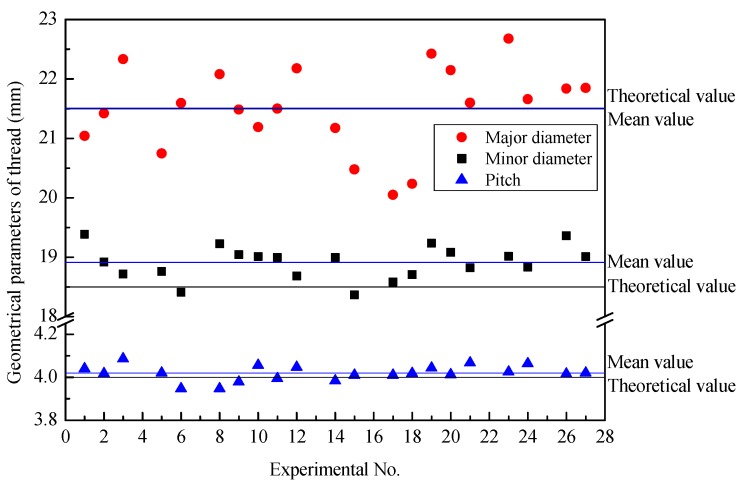
Basis parameters of the threaded section after synchronous rolling.

Figure 11 illustrates the major diameter, minor diameter, and pitch of 21 groups of threaded sections after synchronous rolling. The experimental results fluctuate around the theoretical values. The theoretical value is the desired size of the threaded section, (i.e., the design size of the threaded section). Because the blank diameter is different, the radial feed is slightly different, and the major and minor diameters of the threaded section fluctuate greatly. In particular, the relative error of the minor diameter between the average value and theoretical value is larger, which is 2.2245%. The average value of the major diameter is very close to the theoretical value, and the relative error is 0.0439%.

As shown in Figure 11, there is no obvious influence of the geometrical parameters, billet and motion, on the pitch of the threads of the TSSR die. Under different experimental conditions, the variation of pitch is small, and the fluctuation around theoretical values is also small. The relative error between the average value and theoretical value is 0.4906%. 

According to the data in Table 6 in combination with Equation (6), the primary and secondary effects of each factor can be obtained by range analysis. The interactive column of two factors are two columns, and the one with the greatest range was chosen as the range for the interactive column.

#### 4.1.1. Major Diameter

**Figure 12:**
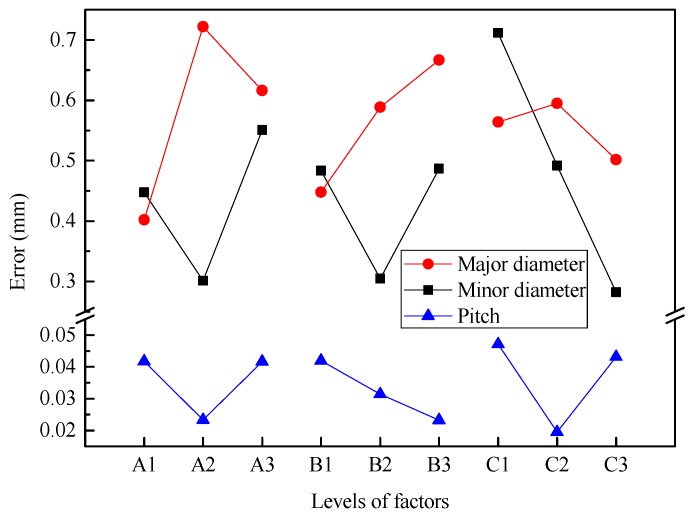
Effect of experimental factors on geometrical error for the threaded section.

**Figure 13:**
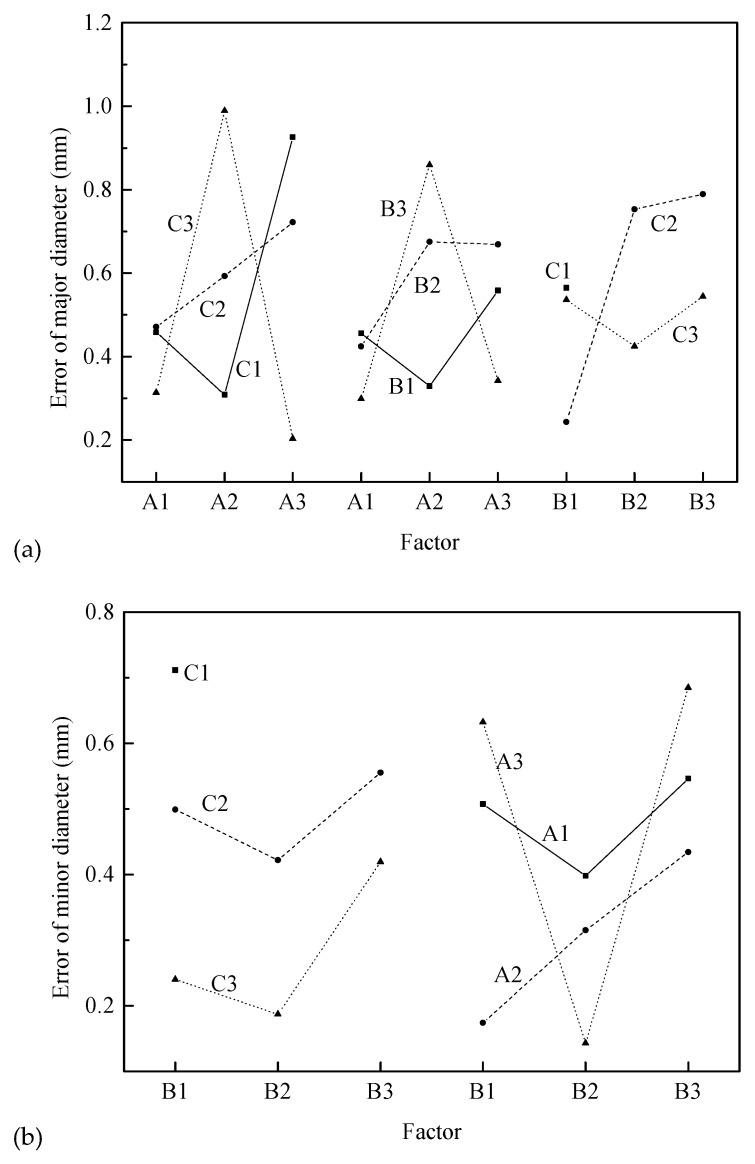

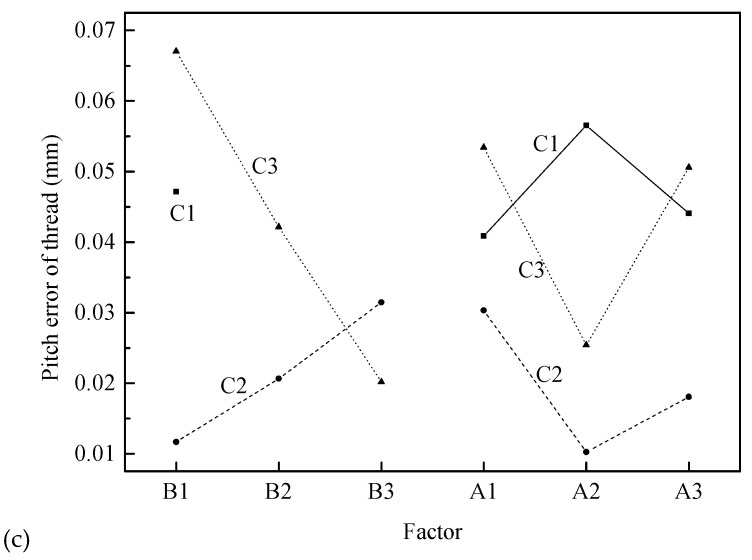
Interactions among factors for the error of geometrical parameters of the threaded section: (**a**) error of major diameter, (**b**) error of minor diameter, (**c**) pitch error of thread.

The range analysis indicates that the order of the factors affecting the error of major/outside diameter for the threaded section is A × C > A × B > B × C ≈ A > B > C. The interaction of factors A and C (i.e., A × C) is the most important factor, the second most important factor is the interaction (A × B) of factors A and B, and then the influence of factors A and interaction B × C is similar and the factors B and C have the smallest range. Considering the influence of interaction, it is necessary to synthetically consider the optimal level according to the trend map (shown in Figure 12) of each factor and the binary effect map (shown in Figure 13a) of interaction.

It can be found from Figure 12 that the error of major diameter increases first and then decreases with increasing billet diameter (factor A), gradually increases with increasing radial feed-in speed (factor B), and first increases and then decreases with increasing rotational speed (factor C).

However, considering the interactions A × C, A × B, and B × C, a comprehensive analyze is needed, according to the information in Figure 12 and Figure 13a. First, for the interaction A × C, A3C3 is the best, but A1C3 and A2C1 are also near the level of A3C3. For the interaction B × C, A1B3 is the best, but A2B1 and A3B3 are also near the level of A1B3. Considering, factor A and interaction B × C, A1B3C3 or A3B3C3 should be chosen. Therefore, the optimum processing conditions are A1B3C3 or A3B3C3, according to the error of major diameter.

#### 4.1.2. Minor Diameter

The range analysis indicates that the order of factors affecting the error of minor diameter for the threaded section is C > A > B > A × B ≈ B × C > A × C. Among them, the influence of factor C is greatest followed by factors A and B. Then the influence of interactions A × B and B × C is almost the same, and the influence of interaction (A × C) of factors A and C is the smallest. Considering the influence of interaction, it is necessary to synthetically consider the optimal level according to the trend map (shown in Figure 12) and the binary effect map (shown in Figure 13b) of interactions A × B and B × C.

Figure 12 illustrates that the error of minor diameter decreases first and then increases with increasing billet diameter (factor A) and radial feed-in speed (factor B), and gradually decreases with increasing rotational speed (factor C).

However, considering the interactions A × B and B × C, a comprehensive analyze is needed, according to the information in Figure 12 and Figure 13b. First, factors C, A, and B should be considered. According to the Figure 12, the best choice is C3A2B2. Then, referring to the binary chart, this choice is acceptable. Therefore, the optimal processing condition is A2B2C3 according to the error of minor diameter.

#### 4.1.3. Pitch of Thread

The range analysis indicates that the order of the factors affecting the pitch error for the threaded section is B × C ≈ C > B ≈ A ≈ A × C > A × B. Among them, the interaction B × C and the factor C are the most important factors, where two factors have almost the same range. The second most important factors are B, A, and interaction A × C, where three factors have almost the same range. Finally, the interaction A × B has the smallest range. Considering the influence of interaction, it is necessary to synthetically analyze the optimal level according to the trend map (shown in Figure 12) and the binary effect map (shown in Figure 13c) of interactions B × C and A × C.

Figure 12 shows that pitch error first decreases and then increases with increasing billet diameter (factor A), gradually decreases with increasing radial feed-in speed (factor B), and first decreases and then increases with increasing rotational speed (factor C).

However, considering interactions B × C and A × C, a synthetical analyze is needed, according to the information in Figure 12 and Figure 13c. First, for interaction B × C and factor C, B1C2 is the best level and for factor A, A2 is the best level combining Figure 12 and Figure 13c. Therefore, the optimum processing condition is A2B1C2 according to the pitch error of thread.

### 4.2. Basis Parameters of Splined Section 

**Figure 14:**
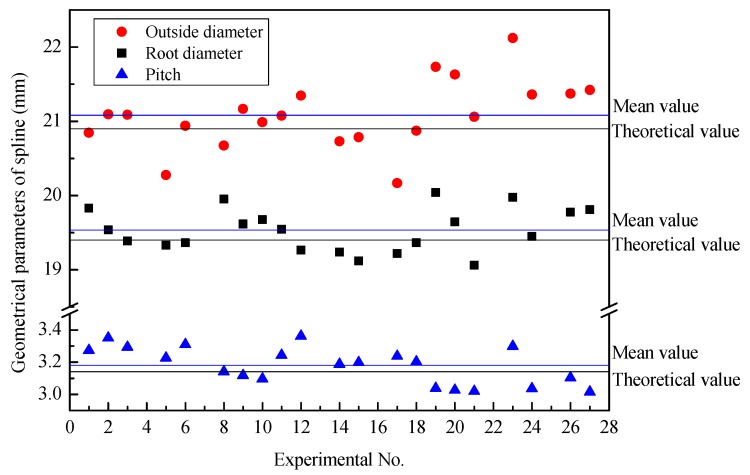
Basis parameters of splined section after synchronous rolling.

Figure 14 illustrates the outside diameter, root diameter, and pitch of 21 groups of splined section after synchronous rolling. The experimental results fluctuate around the theoretical values. The theoretical value is the desired size of the splined section, (i.e., the design size of the splined section). Because the billet diameter is different, the radial feed-in is slightly different, and then the outside diameter, root diameter, and pitch have a large fluctuation. As compared with the parameters of the threaded section, the error between average value and theoretical value is larger for the splined section. The relative errors between the average value and theoretical are larger for outside diameter, root diameter and pitch, being 0.8748%, 0.6856% and 1.2265%, respectively. Among these three basic parameters, the pitch error is the largest. The main reasons for this is that the threaded and splined sections do not coordinate and have a velocity difference, and the meshing of the threaded segments dominates during the synchronous rolling process, and therefore a pitch error of the splined section is produced in order to coordinate deformation [10]. Figure 14 shows that the average values of three basic parameters, such as outside diameter, root diameter and pitch, are slightly larger than the theoretical values.

According to the data in Table 6 in combination with Equation (6), the primary and secondary effects of each factor can be obtained by range analysis. The interactive column of two factor are two columns, and the one with the greatest range was chosen as the range for the interactive column.

#### 4.2.1. Outside Diameter

**Figure 15:**
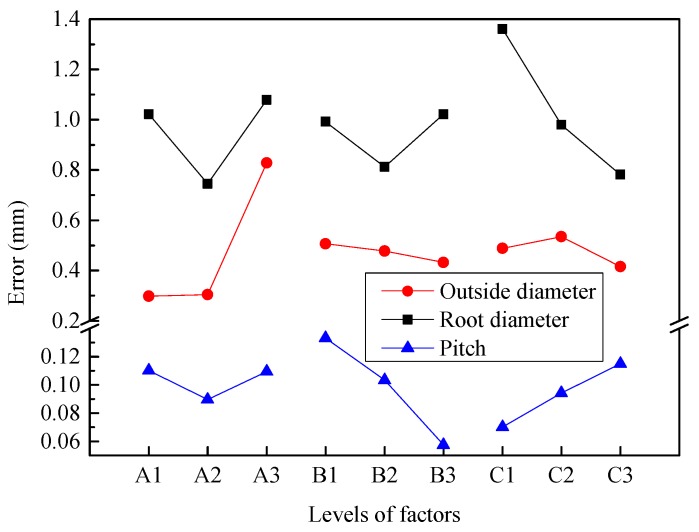
Effect of experimental factors on geometrical error for splined section.

**Figure 16:**
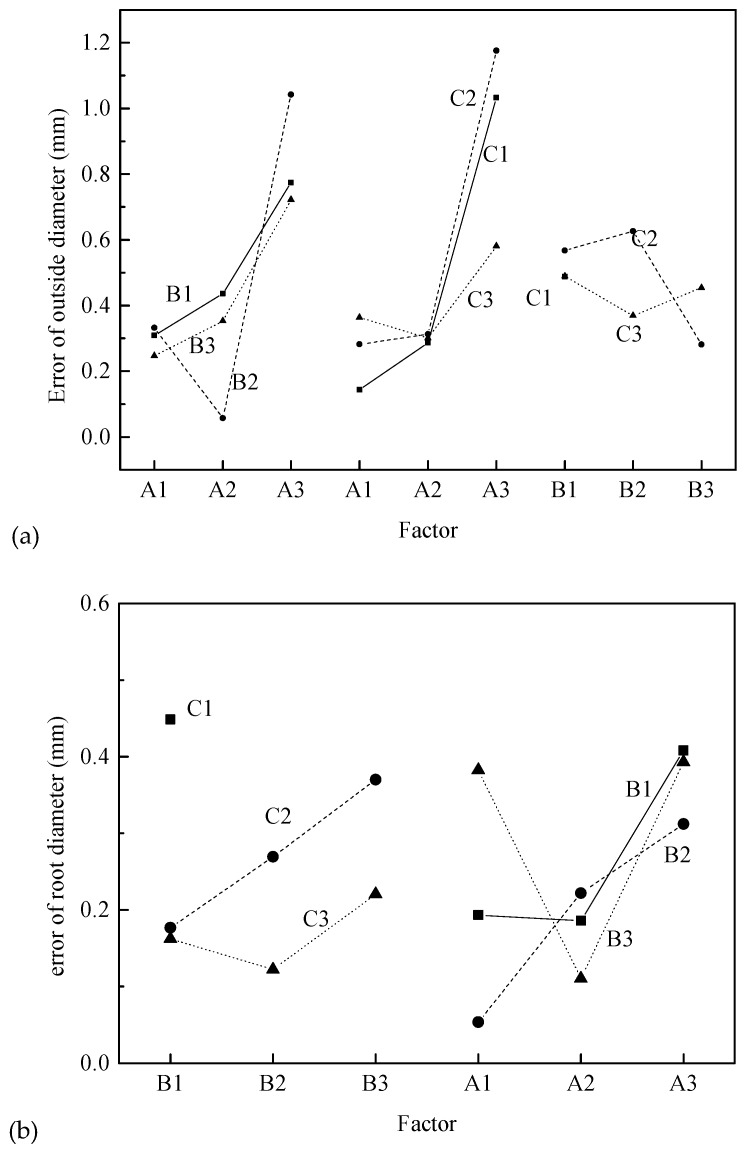

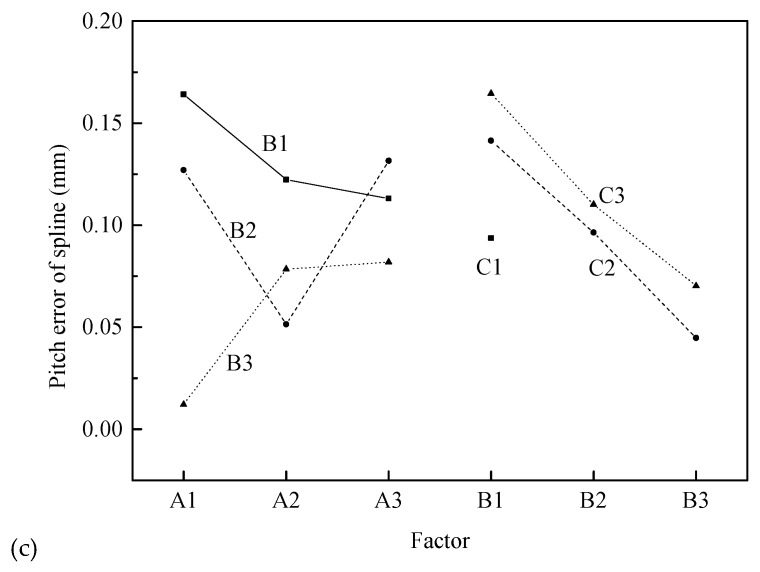
Interactions among factors for the error of geometrical parameters of splined section: (**a**) error of outside diameter, (**b**) error of root diameter, (**c**) pitch error of spline.

The range analysis indicates that the order of the factors affecting the error of outside diameter for the splined section is A > A × B > A × C > B × C > C > B. Among them, the influence of factor A is greatest, followed by interactions A × B, A × C, B × C and factor C, where the influence decreases in turn. The factor B has a small range. Considering the influence of interaction, it is necessary to synthetically consider the optimal level according to the trend map (shown in Figure 15) of each factor and the binary effect map (shown in Figure 16a) of interactions A × B, A × C and B × C.

Figure 15 shows that the error of outside diameter increases smoothly at first and then increases sharply with increasing billet diameter (factor A), gradually decreases with increasing radial feed-in speed (factor B), and first increases and then decreases with increasing rotational speed (factor C).

However, considering the interactions A × B, A × C, B × C, it is necessary to comprehensively analyze according to the information in Figure 15 and Figure 16a. First, factors A, A1, and A2 are almost the same. By considering interaction A × B, level A2B2 is obviously the best. Then considering interactions A × C, B × C and factor C, C3 should be chosen. Therefore, the optimum processing condition is A2B2C3 according to the error of outside diameter.

#### 4.2.2. Root Diameter

The range analysis indicates that the order of factors affecting the error of root diameter for the splined section is C > A > B × C > A × B > B ≈ A × C. The influence of factor C is greatest followed by factor A and interactions B × C and A × B, where the influence decreases in turn. The factor B and interaction A × C have less influence. Considering the influence of interaction, it is necessary to synthetically consider the optimal level according to the trend map (shown in Figure 15) of each factor and the binary effect map (shown in Figure 16b) of interactions B × C and A × B.

Figure 15 shows that the error of root diameter decreases first and then increases with increasing billet diameter (factor A) and radial feed-in speed (factor B), and gradually decreases with increasing rotational speed (factor C).

However, considering the interactions B × C and A × B, it is necessary to comprehensively analyze according to Figure 15 and Figure 16b. First, factor C is the most important factor, and C3 should be selected. Then factor A, and interactions B × C and A × B should be considered comprehensively, and A2B2C3 should be selected. Therefore, the optimal processing condition is A2B2C3 according to the error of the root diameter.

#### 4.2.3. Pitch of Spline

The range analysis indicates that the order of the factors affecting the pitch error for the splined section is B > A × B > B × C ≈ C > A > A × C. The influence of factor B is greatest followed by interaction A × B. Then factor C and interaction B × C follow, and two factors have almost the same range. The factor A and interaction A × C have little influence. Considering the influence of interaction, it is necessary to synthetically analyze the optimal level according to the trend map (shown in Figure 15) and binary effect map (shown in Figure 16c) of interactions A × B and B × C.

Figure 14 shows that the pitch error first decreases and then increases with increasing billet diameter (factor A), gradually decreases with increasing radial feed-in speed (factor B), and gradually increases with increasing rotational speed (factor C). Theoretical study in [10] also showed that the radial feed-in speed of synchronous rolling die has a highly significant effect on the accumulated pitch error of spline, and the feed-in speed can reduce the accumulated pitch error of spline for the synchronous rolling process.

However, considering the interactions B × C and A × B, it is necessary to synthetically analyze according to Figure 15 and Figure 16c. The most important factor is factor B, and then interaction A × B, and therefore A1B3 should be selected. The influence of interaction B × C is almost the same as that of factor C, and therefore B3C2 should be selected. Therefore, the optimum processing condition is A1B3C2 according to the pitch error of the spline.

**Figure 17:**
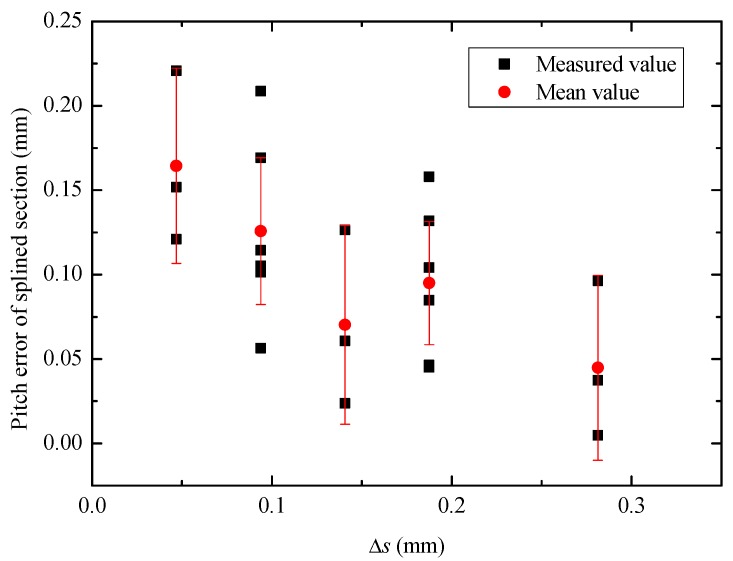
Pitch error of single tooth under different compression amounts.

The compression amount Δs can comprehensively reflect the rotational speed and feed speed of synchronous rolling die. Figure 17 illustrates the pitch error under different compression amounts. It can be seen from Figure 17 that it can ensure the success of synchronous rolling when Δs < 0.3 mm, and the pitch error of single tooth decreases gradually with increases in compression amount, within an appropriate range.

## 5. Conclusions

The effects of billet diameter (*d*), radial feed speed (*v*), and rotational speed (*n_d_*) on the thread and spline synchronous rolling (TSSR) process were studied using an orthogonal experimental design array. Only 21 groups of 27 experiments had successful profiles, and therefore the dimensions of the threaded and splined sections for these 21 experimental workpieces were measured and analyzed. The smallest pitch error of the splined section in the 21 sets of experiments was 0.477 μm, and this indicates that the TSSR process is feasible. On the basis of the experimental results, the following conclusions can be drawn:

(1) For the six failed sets of the TSSR experiment, the thread profile connected well but the spline rolled by different rolling dies did not connect well. This indicated that the meshing motion of the threaded section dominates the rotation during the synchronous rolling process, and uncoordinated motion is more likely to cause tooth error of the splined section of part.

(2) For the TSSR parts, variations of the geometrical parameter of billet and the motion parameters of synchronous rolling die had a greater influence on the size of splined section than that of the threaded section. The geometrical parameter of billet mainly affected the outside diameters of the threaded and splined sections. The motion parameters of synchronous rolling die mainly affected the minor diameter or root diameter and pitch of thread or pitch of spline for the threaded or splined sections, as well the motion parameters of the synchronous rolling die should match each other.

(3) The compression amount (decrement Δs) reflecting the radial deformation degree of the same area during the one rolling unit is a comprehensive indicator of the motion parameters of the synchronous rolling die. The synchronous rolling can proceed smoothly when Δs is less than a certain threshold such as 0.3 mm under the TSSR conditions in this study.

## Figures and Tables

**Table 1:** Three factors with three levels.

Level Rank	*A* ($d_{Z}$ (mm))	*B* (*v* (mm/s))	*C* ($n_{d}$ (r/min))
1	20.00	0.5	8
2	20.25	1.0	16
3	20.50	1.5	32

**Table 2:** Orthogonal experiment design array.

No.	1	2	3	4	5	6	7	8	9	10	11	12	13
	A	B	A × B	A × B	C	A × C	A × C	B × C			B × C		
1	1 (20 mm)	1 (0.5 mm/s)	1	1	1 (8 r/min)	1	1	1	1	1	1	1	1
2	1 (20 mm)	1 (0.5 mm/s)	1	1	2 (16 r/min)	2	2	2	2	2	2	2	2
3	1 (20 mm)	1 (0.5 mm/s)	1	1	3 (32 r/min)	3	3	3	3	3	3	3	3
4	1 (20 mm)	2 (1 mm/s)	2	2	1 (8 r/min)	1	1	2	2	2	3	3	3
5	1 (20 mm)	2 (1 mm/s)	2	2	2 (16 r/min)	2	2	3	3	3	1	1	1
6	1 (20 mm)	2 (1 mm/s)	2	2	3 (32 r/min)	3	3	1	1	1	2	2	2
7	1 (20 mm)	3 (1.5 mm/s)	3	3	1 (8 r/min)	1	1	3	3	3	2	2	2
8	1 (20 mm)	3 (1.5 mm/s)	3	3	2 (16 r/min)	2	2	1	1	1	3	3	3
9	1 (20 mm)	3 (1.5 mm/s)	3	3	3 (32 r/min)	3	3	2	2	2	1	1	1
10	2 (20.25 mm)	1 (0.5 mm/s)	2	3	1 (8 r/min)	2	3	1	2	3	1	2	3
11	2 (20.25 mm)	1 (0.5 mm/s)	2	3	2 (16 r/min)	3	1	2	3	1	2	3	1
12	2 (20.25 mm)	1 (0.5 mm/s)	2	3	3 (32 r/min)	1	2	3	1	2	3	1	2
13	2 (20.25 mm)	2 (1 mm/s)	3	1	1 (8 r/min)	2	3	2	3	1	3	1	2
14	2 (20.25 mm)	2 (1 mm/s)	3	1	2 (16 r/min)	3	1	3	1	2	1	2	3
15	2 (20.25 mm)	2 (1 mm/s)	3	1	3 (32 r/min)	1	2	1	2	3	2	3	1
16	2 (20.25 mm)	3 (1.5 mm/s)	1	2	1 (8 r/min)	2	3	3	1	2	2	3	1
17	2 (20.25 mm)	3 (1.5 mm/s)	1	2	2 (16 r/min)	3	1	1	2	3	3	1	2
18	2 (20.25 mm)	3 (1.5 mm/s)	1	2	3 (32 r/min)	1	2	2	3	1	1	2	3
19	3 (20.5 mm)	1 (0.5 mm/s)	3	2	1 (8 r/min)	3	2	1	3	2	1	3	2
20	3 (20.5 mm)	1 (0.5 mm/s)	3	2	2 (16 r/min)	1	3	2	1	3	2	1	3
21	3 (20.5 mm)	1 (0.5 mm/s)	3	2	3 (32 r/min)	2	1	3	2	1	3	2	1
22	3 (20.5 mm)	2 (1 mm/s)	1	3	1 (8 r/min)	3	2	2	1	3	3	2	1
23	3 (20.5 mm)	2 (1 mm/s)	1	3	2 (16 r/min)	1	3	3	2	1	1	3	2
24	3 (20.5 mm)	2 (1 mm/s)	1	3	3 (32 r/min)	2	1	1	3	2	2	1	3
25	3 (20.5 mm)	3 (1.5 mm/s)	2	1	1 (8 r/min)	3	2	3	2	1	2	1	3
26	3 (20.5 mm)	3 (1.5 mm/s)	2	1	2 (16 r/min)	1	3	1	3	2	3	2	1
27	3 (20.5 mm)	3 (1.5 mm/s)	2	1	3 (32 r/min)	2	1	2	1	3	1	3	2

**Table 3:** Parameters of synchronous rolling die.

Threaded Section			Splined Section		
Parameter	Unit	Value	Parameter	Unit	Value
Pitch diameter	mm	200	Module	mm	1
Pitch	mm	4	Teeth of spline	—	200
Starts of thread	—	10	Pressure angle		45
Half of thread angle		45			

**Table 4:** Chemical constituents of AISI 1045 steel.

	C	Si	Mn	P	S	Cr	Ni	Cu
wt%	0.42~0.50%	0.17~0.37%	0.50~0.80%	≤0.035%	≤0.035%	≤0.25%	≤0.30%	≤0.25%

**Table 5:** Failed workpiece by synchronous rolling.

No.	$d_{Z}$ (mm)	*v* (mm/s)	$n_{d}$ (r/min)	Photo of Failed Workpiece
4	20	1	8	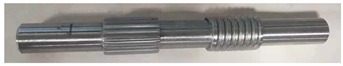
7	20	1.5	8	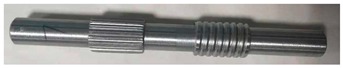
13	20.25	1	8	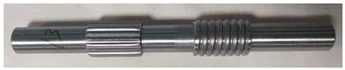
16	20.25	1.5	8	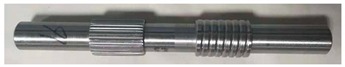
22	20.5	1	8	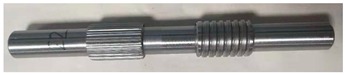
25	20.5	1.5	8	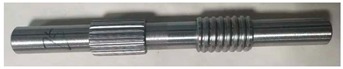

**Table 6:** Measured data (unit: mm).

No.	Threaded Section			Splined Section		
	Major Diameter	Minor Diameter	Pitch	Outside Diameter	Root Diameter	Pitch
1	21.0422	19.3853	4.0409	20.8442	19.8314	3.2733
2	21.4201	18.9179	4.0173	21.0952	19.5378	3.3503
3	22.3316	18.7173	4.0867	21.0866	19.3890	3.2934
4	-	-	-	-	-	-
5	20.7475	18.7574	4.0210	20.2745	19.3302	3.2264
6	21.5946	18.4086	3.9472	20.9393	19.3629	3.3108
7	-	-	-	-	-	-
8	22.0808	19.2226	3.9474	20.6734	19.9513	3.1421
9	21.4837	19.0415	3.9793	21.1668	19.61393	3.1179
10	21.1908	19.0125	4.0565	20.9868	19.67483	3.0965
11	21.4995	18.9975	3.9950	21.0757	19.54573	3.2428
12	22.1765	18.6828	4.0467	21.3448	19.2631	3.3623
13	-	-	-	-	-	-
14	21.1733	18.9931	3.9846	20.7290	19.2369	3.1880
15	20.4775	18.3627	4.0102	20.7856	19.1195	3.1980
16	-	-	-	-	-	-
17	20.0485	18.5816	4.0103	20.1654	19.2169	3.2380
18	20.2333	18.7051	4.0192	20.8717	19.3623	3.2022
19	22.4258	19.2364	4.0441	21.7333	20.0394	3.0376
20	22.1495	19.0812	4.0127	21.6307	19.6463	3.0273
21	21.6000	18.8209	4.0677	21.0592	19.0616	3.0207
22	-	-	-	-	-	-
23	22.6790	19.0141	4.0255	22.1221	19.9752	3.2996
24	21.6583	18.8334	4.0635	21.3612	19.4491	3.0364
25	-	-	-	-	-	-
26	21.8360	19.3610	4.0160	21.3720	19.7760	3.1042
27	21.8495	19.0096	4.0205	21.4219	19.8097	3.0151
